# Eptinezumab treatment was associated with longer interictal headache/migraine periods which corresponded to greater improvements in patient-reported quality of life measures

**DOI:** 10.1007/s00415-024-12809-z

**Published:** 2024-12-12

**Authors:** Stewart J. Tepper, Merle L. Diamond, Joe Hirman, Divya Asher, Damian Fiore, Roger Cady

**Affiliations:** 1 New England Institute for Neurology and Headache, Stamford, CT USA; 2 Diamond Headache Clinic, Chicago, IL USA; 3 Pacific Northwest Statistical Consulting, Inc, Woodinville, WA USA; 4 Lundbeck LLC, Deerfield, IL USA; 5 RK Consults, Ozark, MO USA; 6 Missouri State University, Springfield, MO USA; 7 Axon Therapeutics, San Diego, CA USA

**Keywords:** Chronic migraine, Eptinezumab, Preventive migraine treatment, Patient-reported outcomes, Quality of life

## Abstract

**Introduction:**

Longer periods between headache episodes (interictal periods) may provide greater time for the nervous system to reset from a previous episode, potentially improving disease status and health-related quality of life. This post hoc analysis evaluated this hypothesis by associating patients’ longest interictal periods with improvements in patient-reported outcomes.

**Methods:**

PROMISE-2 (NCT02974153) was a double-blind, placebo-controlled study evaluating eptinezumab for preventive treatment of chronic migraine (N = 1072). Daily electronic diary data from Weeks 1–12 and Weeks 1–24 were used to identify interictal periods, defined as days between headache episodes. For each patient, the longest interictal period within these intervals was identified and categorized (1–4, 5–9, 10–14, > 14, and > 21 days). For each category, the following patient-reported outcomes were assessed: 6-item Headache Impact Test (HIT-6), Patient Global Impression of Change (PGIC), and patient-identified most bothersome symptom (PI-MBS).

**Results:**

Excluding interictal periods with > 10% missing data (resulting in 1010 patients with sufficient data), the mean (SD) of longest interictal periods over Weeks 1–12 was 9.4 (11.0) days. A ≥6-point HIT-6 reduction was observed in 78% (56/72) vs 26% (91/351) of patients with a > 21-day vs 1–4-day longest interictal period, respectively; much or very much improvement per PGIC was reported in 90% (65/72) vs 25% (87/348), respectively, and per PI-MBS was reported in 88% (63/72) vs 26% (92/348), respectively. Similar results were observed for Weeks 1–24.

**Conclusion:**

Longer interictal periods were associated with more patients indicating positive changes in headache-related life impact, disease status, and symptomology.

**Trial registration:** ClinicalTrials.gov (identifier: NCT02974153; registered: 2016-11-23)

**Supplementary Information:**

The online version contains supplementary material available at 10.1007/s00415-024-12809-z.

## Introduction

Migraine is one of the leading causes of years lost to disability globally [1]. Migraine is a complex neurobiological process characterized by numerous potential symptoms such as headache, sensory sensitivity, nausea/vomiting, autonomic symptomatology, muscle pain, cognitive impairment, incoordination, and fatigue. Migraine typically recurs at varying frequencies over decades of a person’s life with individual migraine attacks lasting a few hours to more than 7 days [2, 3]. Some patients with migraine have their disease progress into chronic migraine (i.e., headache on ≥ 15 days/month for > 3 months [3]), which can lead to enduring migraine and headache symptoms on most days of the month [4].

The period in between headache episodes, including migraine attacks, is referred to as the “interictal period” [5]. Although patients may experience lingering symptoms between headache episodes, the interictal period is generally a time of neurological stability and paucity of migraine symptomatology relative to the time during a headache episode [6, 7]. It has been hypothesized that having longer interictal periods can give the nervous system more time to recover from previous episodes, potentially bolstering psychological resiliency and possibly increasing the threshold to future headache episodes, including migraine. This post hoc analysis was performed to evaluate this hypothesis by determining if longer interictal periods are associated with greater improvements in patient-reported outcome measures than shorter interictal periods; data were from PROMISE-2, a clinical trial that studied the efficacy and safety of eptinezumab, a monoclonal antibody that targets the calcitonin gene-related peptide (CGRP) ligand, for the preventive treatment of chronic migraine in adults [8].

## Methods

### Study design

PROMISE-2 was a double-blind, randomized, placebo-controlled pivotal study that evaluated the efficacy and safety of eptinezumab for preventive treatment of migraine in adults with chronic migraine. This study is registered on ClinicalTrials.gov (NCT02974153). The full study protocol and statistical analysis plan have been published [9].

Adults with chronic migraine who met all inclusion criteria (18–65 years of age [inclusive]; a diagnosis of migraine at ≤ 50 years of age; history of chronic migraine for ≥ 12 months; and experienced ≥ 15 to ≤ 26 headache days and ≥ 8 migraine days during screening) were randomized to receive eptinezumab 100 mg, eptinezumab 300 mg, or placebo via intravenous infusion. As eptinezumab has a half-life of approximately 4 weeks [10], study intervention was administered on Day 0 and at Week 12. To assess study outcomes in PROMISE-2, patients completed an electronic diary (eDiary) on a daily basis independent of headache occurrence as well as a separate report entry for each headache. Within this diary, the patient reported the start and end of their headache episodes as well as symptoms experienced and acute medication use. This post hoc analysis explored PROMISE-2 eDiary data from Weeks 1–12 and Weeks 1–24 to identify headache-free (i.e., interictal) periods.

### Patient-reported outcome measures

The 6-item Headache Impact Test (HIT-6) assesses the impact of headache on a patient’s ability to function [11]. HIT-6 is a Likert-type, self-reporting questionnaire composed of 6 questions. Each question is rated from “never” to “always,” with the following scores: never = 6, rarely = 8, sometimes = 10, very often = 11, and always = 13. The total score is the sum of responses and can range from 36 to 78. Scores ≥ 60 signify severe impact; 56‒59, substantial impact; 50‒55, some impact, and ≤ 49 little to no impact. A reduction ranging from 3‒5 in HIT-6 total score has been shown to be clinically significant across migraine literature [12]. A reduction of ≥6 points in HIT-6 total score has been shown to be clinically significant in patients with chronic migraine (total score responders), with HIT-6 item improvements of ≥ 1 or ≥ 2 categories considered clinically meaningful depending on the item (item responders) [12]. HIT-6 was captured in PROMISE-2 at screening, Day 0 (baseline), and at Weeks 4, 12, 16, 24, and 32.

The Patient Global Impression of Change (PGIC) assesses a patient’s perspective on whether they feel their disease has improved, worsened, or remained the same compared to the start of study [13]. The PGIC is a single question with 7 possible answers that range from “very much worse” to “very much improved.” As a measure of change, PGIC was captured in PROMISE-2 post-baseline at Weeks 4, 8, 12, 16, 20, 24, and 32. PGIC scores correlate strongly with the patient-identified most bothersome symptom (PI-MBS) measure [14, 15], which is described below.

The PI-MBS measure is a migraine-specific individualized questionnaire in which patients self-selected their most bothersome migraine-related symptom [14]. In PROMISE-2, patients verbally described their most bothersome migraine-associated symptom at the screening visit. After screening, patients were asked about the improvement in their PI-MBS, with responses, as with the PGIC, ranging from “very much worse” to “very much improved.” Similar to PGIC, PI-MBS was captured at Weeks 4, 8, 12, 16, 20, 24, and 32. As noted above, patient improvement in their PI-MBS has a high statistical correlation with the patient’s PGIC [14, 15].

### Statistical analysis

As a post hoc analysis, no power calculations were performed, and all results are descriptive. An interictal period was defined as the days after the end of one headache and the start of the next headache as reported in the eDiary. The boundaries for interictal periods varied depending on the time period assessed (e.g., Weeks 1–12 or 1–24). For headache-free periods starting prior to baseline treatment (i.e., the patient was headache-free on Day 0), the interictal period was allowed to include the pre-treatment days. For interictal periods ongoing at the Week 12 visit, the length was terminated at the Week 12 visit date for the Weeks 1–12 analyses, and for interictal periods ongoing at the Week 24 visit, the length was terminated at the Week 24 visit date for the Weeks 1–24 analyses. Patients who did not attend the Week 12 visit were not included in the Weeks 1–12 analyses, and patients who did not attend the Week 24 visit were not included in the Weeks 1–24 analyses.

For these analyses, any intervals with > 10% of eDiary days missing were excluded. For each patient, the longest interictal period was identified and used for the analysis of patient-reported outcomes. Treatment arms (eptinezumab 100 mg, eptinezumab 300 mg, or placebo) were analyzed in combination to show the effect of interictal periods for the migraine population in general. For the Weeks 1–12 analyses the patient-reported outcomes are the results reported by the patient at Week 12, and similarly for the Weeks 1–24 analyses the results are based upon what the patient reported at Week 24. All analyses were performed using SAS software v9.4 (SAS Institute, Inc., Cary, NC, USA).

## Results

### Population and distribution

In PROMISE-2, a total of 1072 patients received treatment and were included in the full analysis population. Demographic and baseline clinical characteristics were summarized in the main publication [8]. In brief, the mean age was 40.5 years, and most patients were female (88.2%) and White (91.0%). The mean duration from the time of migraine diagnosis was 18.1 years, and the total population had an average of 20.5 headache days per month. Mean HIT-6 total score was 65 across groups, indicating severe headache-related life impact. The most commonly reported PI-MBS was light sensitivity (19%) [14].

**Fig. 1:**
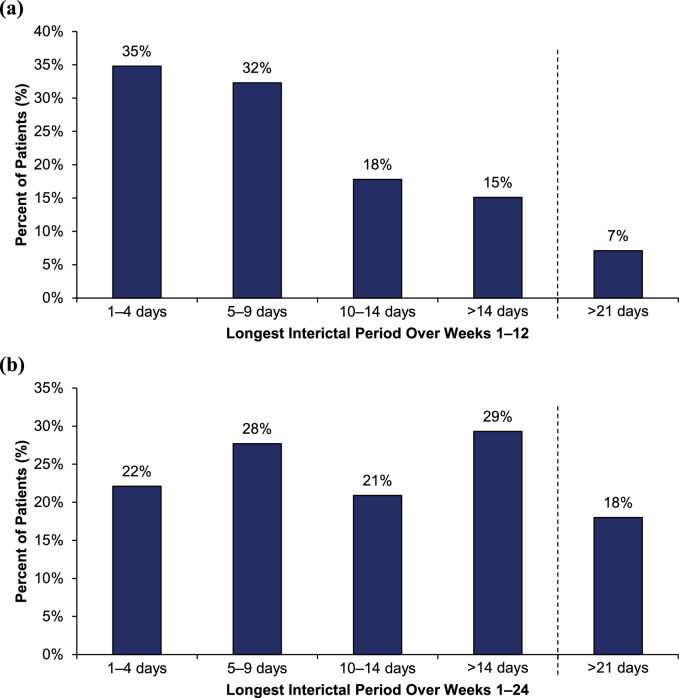
Longest interictal period over **a** Weeks 1‒12 (N = 1010) and **b** Weeks 1‒24 (N = 972). All treatment arms (eptinezumab 100 mg, 300 mg, and placebo) were pooled for analysis

Analysis of interictal periods over Weeks 1‒12 identified 13,854 interictal periods (4651 with eptinezumab 100 mg, 4380 with eptinezumab 300 mg, and 4823 with placebo) across 1026 patients in the study at Week 12. The mean (standard deviation [SD]) length of interictal period was 5.3 (7.7) days (5.0 [4.8] for eptinezumab 100 mg, 6.6 [10.6] days for eptinezumab 300 mg, and 4.4 [6.4] days for placebo). Excluding periods with > 10% missing data (resulting in 1010 patients with sufficient periods), the mean (SD) of the longest interictal period per patient was 9.4 (11.0) days (9.3 [10.1] for eptinezumab 100 mg, 11.5 [13.9] days for eptinezumab 300 mg, and 7.5 [8.0] days for placebo). The longest interictal period over Weeks 1–12 in about two-thirds of patients were in two groups: 1–4 days in 35% and 5–9 days in 32% (pooled population, Fig. 1A; individual treatment arms, Supplementary Fig. 1A).

In the analysis over Weeks 1‒24, there were 23,643 interictal periods identified (7855 with eptinezumab 100 mg, 7399 with eptinezumab 300 mg, and 8389 with placebo) across 983 patients in the study at Week 24. Mean length of interictal periods over Weeks 1–24 was longer than over Weeks 1–12. Across all Weeks 1–24 interictal periods, the mean (SD) length was 6.9 (11.2) days (6.6 [7.9] for eptinezumab 100 mg, 8.6 [16.0] days for eptinezumab 300 mg, and 5.4 [7.2] days for placebo). Excluding periods with > 10% missing data (resulting in 972 patients with sufficient periods), the longest mean (SD) interictal period was 15.0 (18.9) days in the total population (15.6 [20.0] for eptinezumab 100 mg, 17.8 [22.1] days for eptinezumab 300 mg, and 11.5 [13.0] days for placebo). Unlike over Weeks 1–12, the distribution was bimodal in the total population, peaking at 5–9 days and > 14 days (pooled population, Fig. 1B; individual treatment arms, Supplementary Fig. 1B). Across Weeks 13–24 specifically, there were 10,958 interictal periods reported across 979 patients; excluding periods with > 10% missing data resulted in 909 patients with sufficient periods for analysis. The mean (SD) length of the longest interictal periods was 14.0 [19.2] days (15.6 [21.3] for eptinezumab 100 mg and 300 mg combined and 10.7 [13.4] days for placebo). The distribution over Weeks 13–24 was bimodal, similar to the distribution over Weeks 1–24.

### Patient-reported outcome measures

**Fig. 2:**
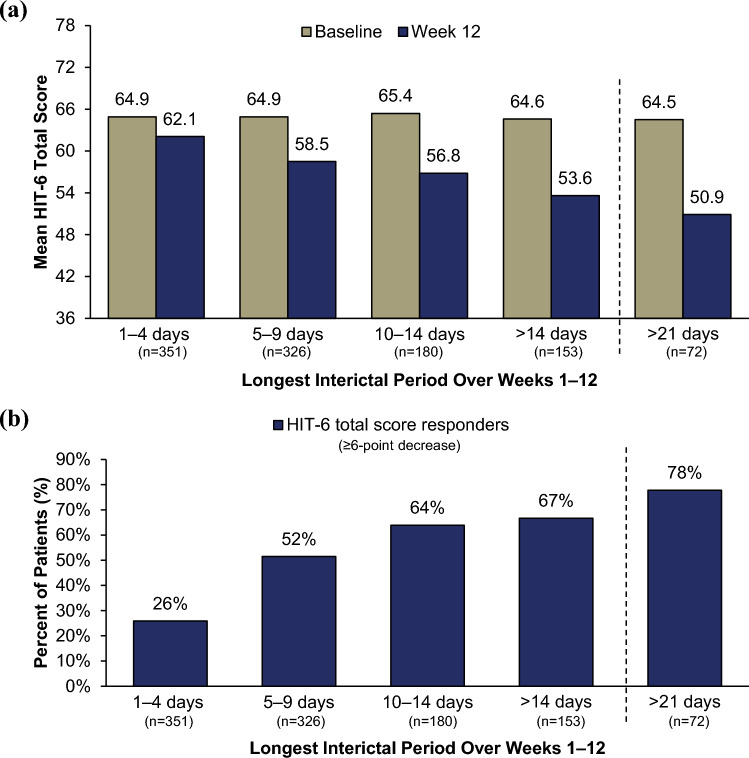
HIT-6 **a** total score and **b** responder rates by longest interictal period over Weeks 1‒12. All treatment arms (eptinezumab 100 mg, 300 mg, and placebo) were pooled for analysis. HIT-6, 6-item Headache Impact Test

Patients were categorized by their longest interictal period, and changes in HIT-6 total score from baseline to Week 12 were analyzed. Longer periods between headache were associated with greater changes in HIT-6 total score, with a mean change of –11.0 and –13.6 in patients with longest intervals > 14 days and > 21 days, respectively (Fig. 2A). Mean change from baseline in HIT-6 total score suggested a clinically meaningful change (≥ 6-point decrease) was observed for the patients with interictal periods as short as 5–9 consecutive days (–6.4 average change in HIT-6 total score). The percentage of HIT-6 total score responders (≥ 6-point decrease) was > 60% for patients with a longest interictal period of > 10 days (Fig. 2B). A similar trend was observed when analyzing headache-free periods over Weeks 1–24 (Supplementary Fig. 2). As with HIT-6 total score responders, longer interictal periods were associated with a higher percentage of HIT-6 item responders; this trend was consistent across all items (HIT-6 items 1–3, Supplementary Fig. 3A; HIT-6 items 4–6, Supplementary Fig. 3B).

**Fig. 3:**
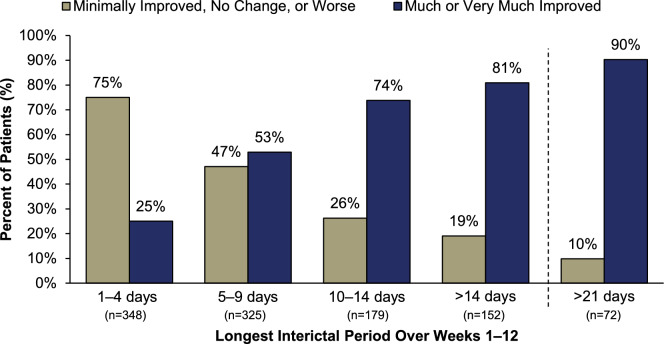
PGIC rating by longest interictal period over Weeks 1‒12. All treatment arms (eptinezumab 100 mg, 300 mg, and placebo) were pooled for analysis. PGIC, Patient Global Impression of Change

**Fig. 4:**
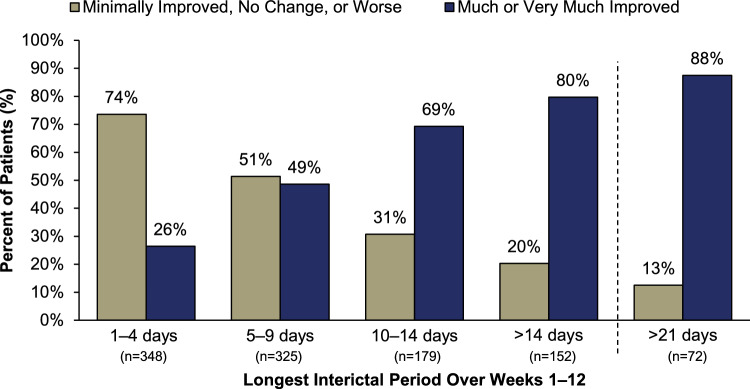
PI-MBS rating by longest interictal period over Weeks 1‒12. All treatment arms (eptinezumab 100 mg, 300 mg, and placebo) were pooled for analysis. PI-MBS, patient-identified most bothersome symptom

Analysis of PGIC rating by patients’ longest interictal period revealed comparable findings. As the interictal periods lengthened, the percentage of patients selecting “much improved” or “very much improved” increased substantially. By Week 12, 90% of patients with a > 21-day longest interictal period reported “much” or “very much” improved (Fig. 3). When interictal periods reached > 10 days to > 21 days, 69–88% of patients reported their PI-MBS was “much” or “very much” improved (Fig. 4). A similar trend was observed when analyzing interictal periods over Weeks 1–24 (Supplementary Figs. 4 and 5, respectively).

## Discussion

Most analyses from clinical trials in migraine focus on disease symptomatology and burden. This post hoc analysis assessed duration of interictal periods and evaluated for sustained improvement in HIT-6, PI-MBS, and PGIC to assess the hypothesis that longer periods between headache, these interictal periods, lessen the disease impact of migraine and increase the patient’s perception of positive change in disease over time. Consistent with the hypothesis, across all patient-reported outcome measures, longer interictal periods were associated with greater patient self-reported improvement.

It is important to note that for this analysis interictal periods are patient-reported headache-free periods derived from an eDiary, and therefore patients may not be completely free of all headache or migraine symptoms. Despite this, there were dramatic improvements observed. Clinically meaningful changes in the HIT-6 (≥ 6-point decrease [12]) were observed with interictal periods as short as 5–9 days (–6.4-point change), and more than 60% of HIT-6 total score responders (≥ 6-point decrease [12]) were observed when the interictal period was > 10 days. Analysis of PGIC rating and PI-MBS by longest interictal period revealed similar findings, and as the interictal periods lengthened, the percentage of patients selecting “much or very much improved” increased substantially. It is worth repeating that while the PGIC is only 1 question, the response to this question correlates with other major outcome measures, including the HIT-6 and PI-MBS, as observed in this study [14, 15]. Thus, both PGIC and PI-MBS are reasonable evaluations of the success of treatment on the overall migraine effect on a person with migraine, and improvements per those measures suggest a pervasive benefit associated with longer interictal headache-free periods.

While the goal of this study was to observe the general chronic migraine population, when analyzing individual arms from the PROMISE-2 study, the data showed that patients treated with eptinezumab (100 mg or 300 mg) had longer interictal periods than those receiving placebo. Over Weeks 1‒12, the longest mean interictal period was 9.3 days for eptinezumab 100 mg and 11.5 days for eptinezumab 300 mg, compared to 7.5 days for placebo, and mean length of interictal periods over Weeks 1–24 was longer than over Weeks 1–12, suggesting continued improvement over time. There were also consistently numerically longer interictal periods with the higher eptinezumab dosage of 300 mg than 100 mg or placebo.

Across several clinical trials, eptinezumab treatment demonstrated efficacy at reducing the frequency of monthly migraine days [8, 16–19], to which increasing the length of the interictal burden may be considered simply the inverse. However, analyses demonstrate that the effect of eptinezumab is not limited to reducing migraine frequency and includes reducing the frequency of severe migraine attacks, improving PI-MBS, improving other migraine-related symptoms, and providing benefit on functioning and health-related quality of life [8, 14, 18–24]. Given the relationships between interictal period length and patient-reported outcomes demonstrated in the current analysis, measuring the number of days between active headache may provide more insight into the effects of preventive treatment than solely the reduction in migraine frequency. A recent real-world study of eptinezumab in patients with chronic migraine asked patients about the number of “good days” per month, which was undefined in the survey, and the number of “good days” per month more than doubled after starting eptinezumab treatment [25].

The clinical implications that “neurological rest” between attacks may be an important component in overall health-related quality of life that is often overlooked or underappreciated in clinical practice [26]. These results suggest that longer headache-free periods provide the nervous system more time to recover, which in turn leads to a measurable change in a patient’s quality of life and migraine symptomology and can help provide more clinical insight into the measurable benefits of “neurological rest” for patients with migraine.

### Limitations

This study has some limitations beyond those inherent to post hoc analyses. The analyses only focused on patient-reported headache days and did not focus on other symptoms that may have been present during the premonitory, postdromal, or interictal periods [2, 27]. The influence of the burden during premonitory and postdromal periods and during non-headache days could not be incorporated, which may have impacted the effect of longer interictal periods on quality of life measures. Analyses conducted using data pooled from all three treatment arms (eptinezumab 100 mg, 300 mg, and placebo) did not account for the influence of treatment effect. Lastly, the PROMISE-2 trial population was mostly female and White, and overall results may not be generalizable to the full migraine population.

## Conclusions

In this analysis, longer interictal periods between headache (including migraine) were associated with positive changes in patients’ perception of their health-related quality of life as measured by the patient-reported HIT-6, PGIC, and PI-MBS measures. The hypothesis underscoring this analysis is that success begets success and that longer periods without headache/migraine potentially reflects better levels of physiological migraine recovery. In turn, the threshold to future migraine attacks may increase and be synergistic or additive with preventive medications and behaviors, supported in this data set by the observation that longer interictal periods, regardless of being on drug or placebo, were associated with marked improvement in patient-reported outcome measures. Duration of interictal periods was numerically longer with eptinezumab than placebo, numerically longer with the higher dose of eptinezumab 300 mg than 100 mg, and numerically longer over time with repeated eptinezumab doses. Results presented here highlight the need for clinical goals to focus not only on the overall decrease in frequency of monthly migraine attacks, but also lengthening the time between these attacks, thereby increasing the interictal periods and potentially improving overall quality of life. Migraine-specific preventive treatments can play a part in this.

## Supplementary Information

Below is the link to the electronic supplementary material.

Supplementary file1 (PDF 273 KB)

## Data Availability

In accordance with EFPIA’s and PhRMA’s “Principles for Responsible Clinical Trial Data Sharing” guidelines, Lundbeck is committed to responsible sharing of clinical trial data in a manner that is consistent with safeguarding the privacy of patients, respecting the integrity of national regulatory systems, and protecting the intellectual property of the sponsor. The protection of intellectual property ensures continued research and innovation in the pharmaceutical industry. Deidentified data are available to those whose request has been reviewed and approved through an application submitted to https://www.lundbeck.com/global/our-science/clinical-data-sharing.
