# Supplementary material for: Eptinezumab treatment was associated with longer interictal headache/migraine periods which corresponded to greater improvements in patient-reported quality of life measures
Source: J Neurol. 2024 Dec 12;272(1):4. doi: 10.1007/s00415-024-12809-z (PMC11638385; doi:10.1007/s00415-024-12809-z)
Supplement: Supplementary file 1 — Supplementary file1 (PDF 273 KB) [file 415_2024_12809_MOESM1_ESM.pdf]

SUPPLEMENTARY INFORMATION

Journal of Neurology

Eptinezumab treatment was associated with longer interictal headache/migraine periods which corresponded to greater improvements in patient-reported quality of life measures

Stewart J. Tepper[1], Merle L. Diamond[2], Joe Hirman[3], Divya Asher[4], Damian Fiore[4], Roger Cady[5-7]

[1]*New England Institute for Neurology and Headache, Stamford, Connecticut, United States;* [2]*Diamond Headache Clinic, Chicago, Illinois, United States;* [3]*Pacific Northwest Statistical Consulting, Inc., Woodinville, Washington, United States;* [4]*Lundbeck LLC, Deerfield, Illinois, United States;* [5]*RK Consults, Ozark, Missouri, United States;* [6]*Missouri State University, Springfield, Missouri, United States;* [7]*Axon Therapeutics, San Diego, California, United States*

Corresponding Author: Stewart J. Tepper, sjtepper@gmail.com

Supplementary Fig. 1 Longest interictal period over **(a)** Weeks 1–12 and **(b)** Weeks 1–24 by treatment arm

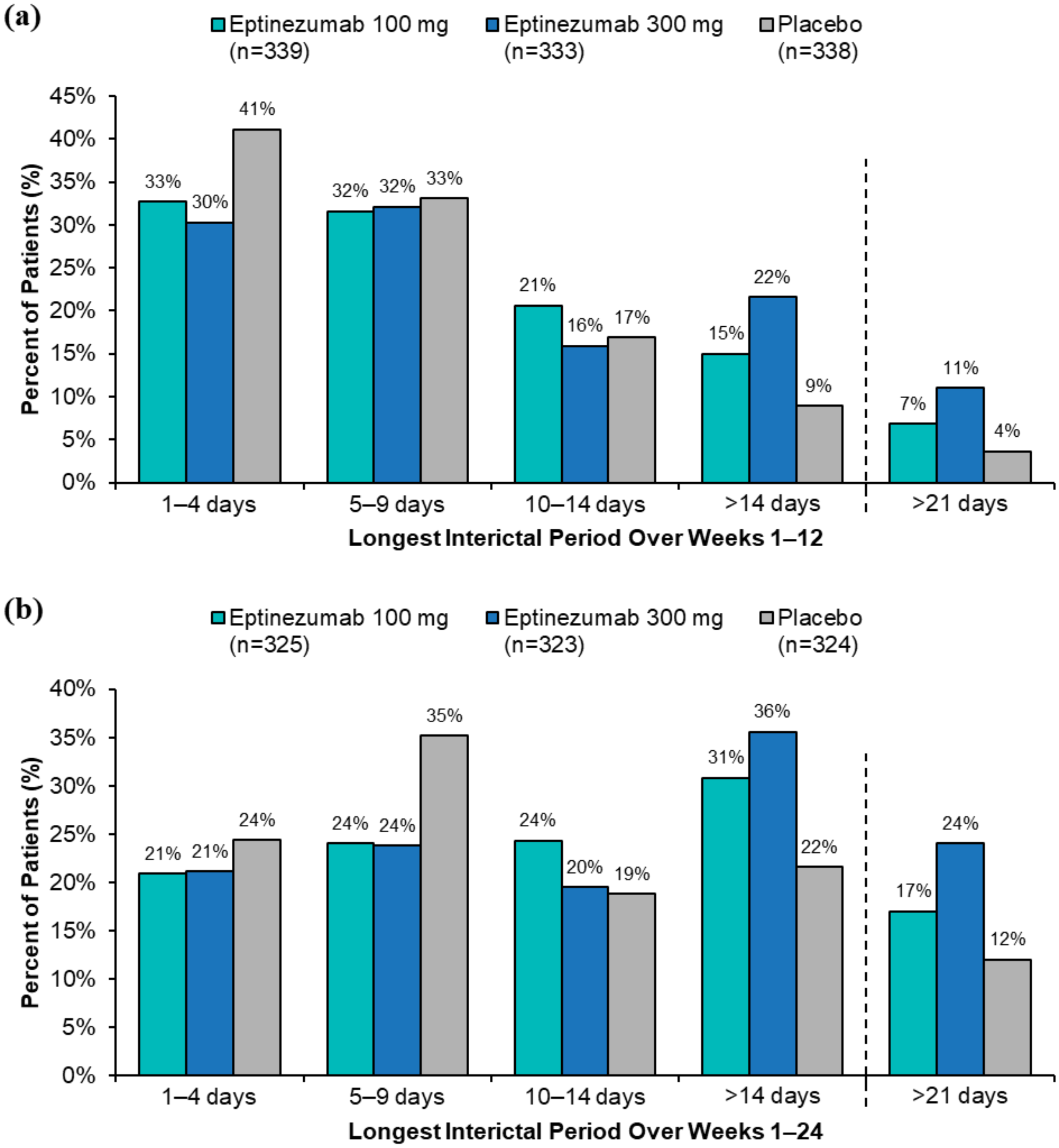

Supplementary Fig. 2 HIT-6 total score by longest interictal period over Weeks 1–24

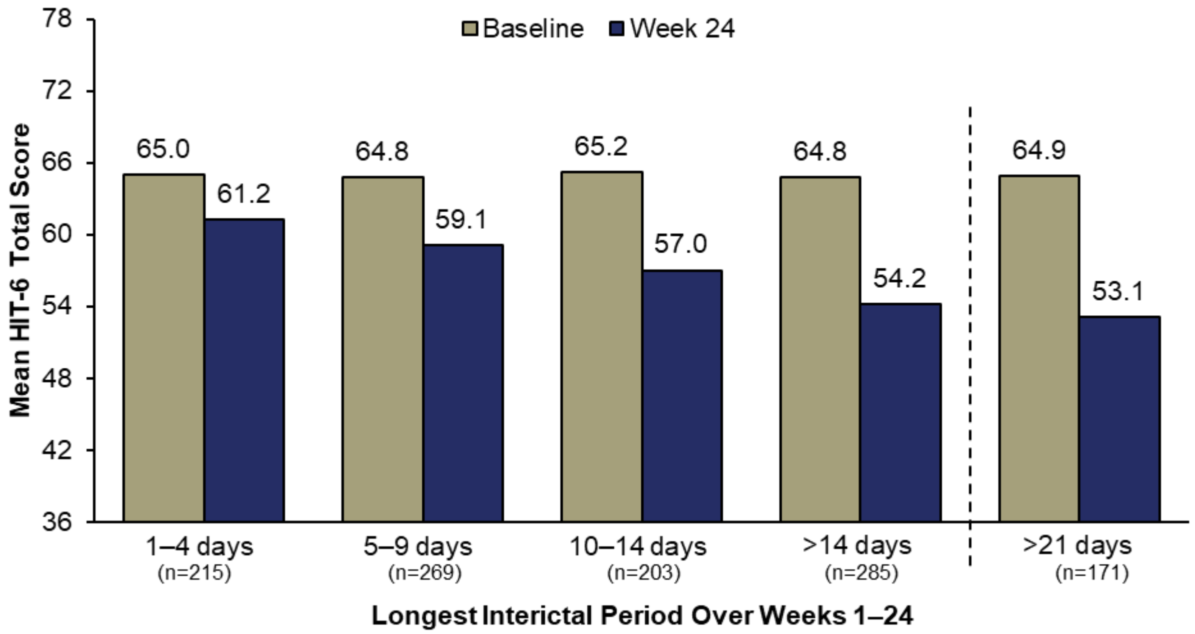

All treatment arms (eptinezumab 100 mg, 300 mg, and placebo) were pooled for analysis.

HIT-6, 6-item Headache Impact Test.

Supplementary Fig. 3 HIT-6 responders on **(a)** items 1–3 [1-category improvement] and **(b)** items 4–6 [2-category improvement] by longest interictal period over Weeks 1–12

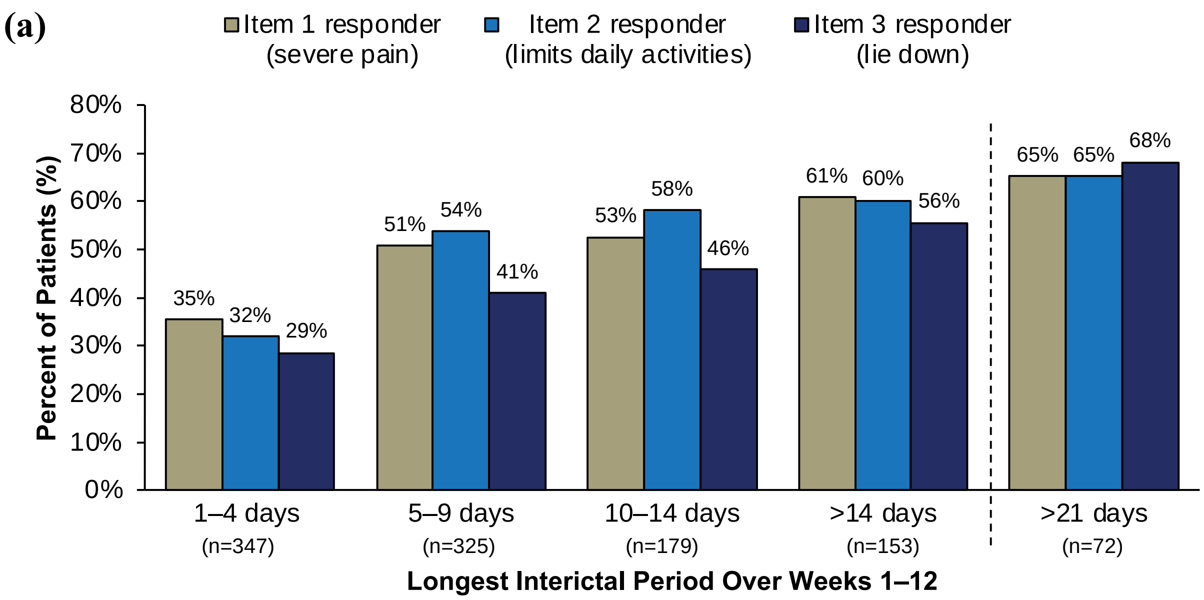

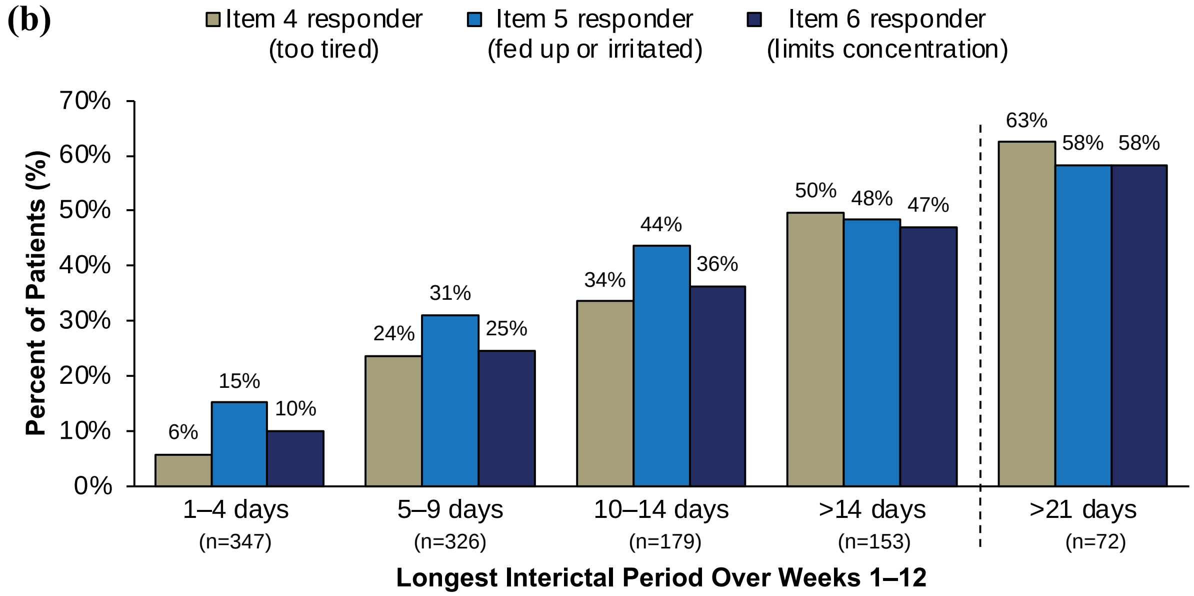

All treatment arms (eptinezumab 100 mg, 300 mg, and placebo) were pooled for analysis.

HIT-6, 6-item Headache Impact Test.

Supplementary Fig. 4 PGIC rating by longest interictal period over Weeks 1–24

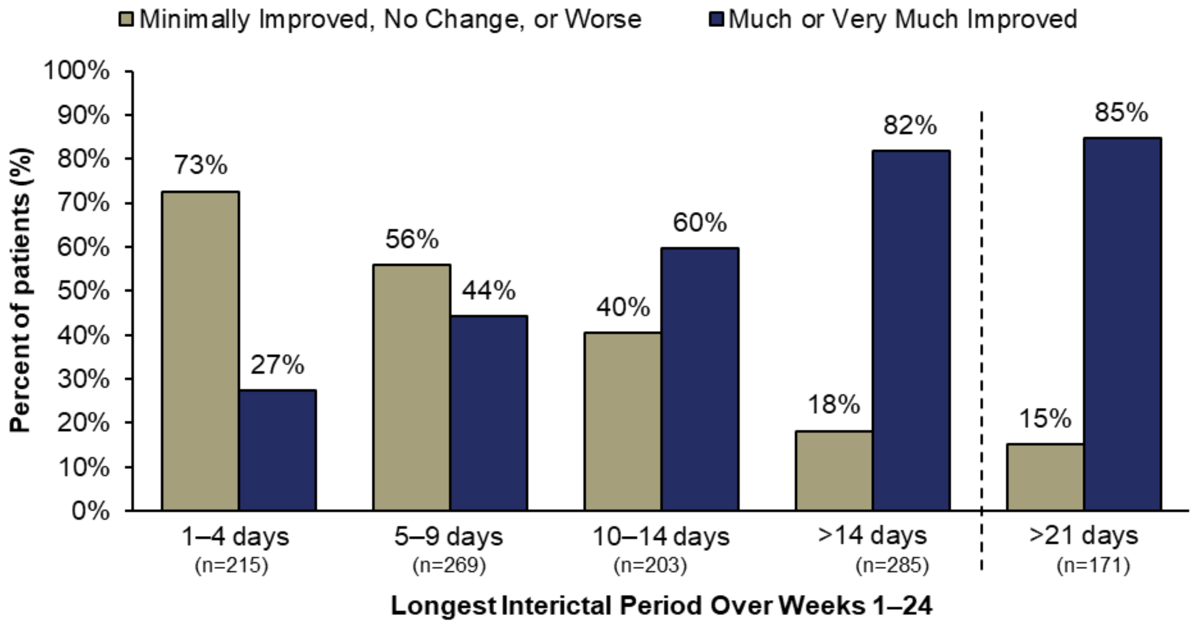

All treatment arms (eptinezumab 100 mg, 300 mg, and placebo) were pooled for analysis.

Percentages may not sum to 100 due to rounding.

PGIC, Patient Global Impression of Change.

Supplementary Fig. 5 PI-MBS rating by longest interictal period over Weeks 1–24

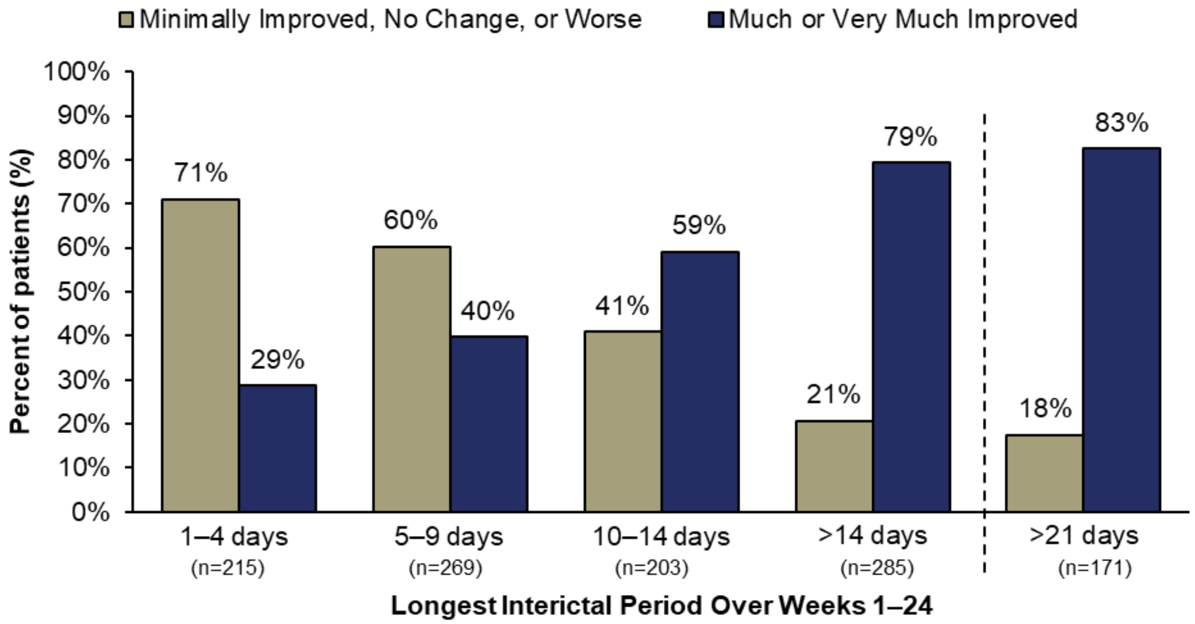

All treatment arms (eptinezumab 100 mg, 300 mg, and placebo) were pooled for analysis.

Percentages may not sum to 100 due to rounding.

PI-MBS, Patient-identified most bothersome symptom.
